# The Relation Between Capillary Transit Times and Hemoglobin Saturation Heterogeneity. Part 1: Theoretical Models

**DOI:** 10.3389/fphys.2018.00420

**Published:** 2018-04-26

**Authors:** Adrien Lücker, Timothy W. Secomb, Bruno Weber, Patrick Jenny

**Affiliations:** ^1^Department of Mechanical and Process Engineering, Institute of Fluid Dynamics, ETH Zurich, Zurich, Switzerland; ^2^Department of Physiology, University of Arizona, Tucson, AZ, United States; ^3^Institute of Pharmacology and Toxicology, University of Zurich, Zurich, Switzerland

**Keywords:** blood flow, capillary transit time heterogeneity, computational modeling, hematocrit, hemoglobin saturation, microcirculation, oxygen transport, red blood cells

## Abstract

Capillary dysfunction impairs oxygen supply to parenchymal cells and often occurs in Alzheimer's disease, diabetes and aging. Disturbed capillary flow patterns have been shown to limit the efficacy of oxygen extraction and can be quantified using capillary transit time heterogeneity (CTH). However, the transit time of red blood cells (RBCs) through the microvasculature is not a direct measure of their capacity for oxygen delivery. Here we examine the relation between CTH and capillary outflow saturation heterogeneity (COSH), which is the heterogeneity of blood oxygen content at the venous end of capillaries. Models for the evolution of hemoglobin saturation heterogeneity (HSH) in capillary networks were developed and validated using a computational model with moving RBCs. Two representative situations were selected: a Krogh cylinder geometry with heterogeneous hemoglobin saturation (HS) at the inflow, and a parallel array of four capillaries. The heterogeneity of HS after converging capillary bifurcations was found to exponentially decrease with a time scale of 0.15–0.21 s due to diffusive interaction between RBCs. Similarly, the HS difference between parallel capillaries also drops exponentially with a time scale of 0.12–0.19 s. These decay times are substantially smaller than measured RBC transit times and only weakly depend on the distance between microvessels. This work shows that diffusive interaction strongly reduces COSH on a small spatial scale. Therefore, we conclude that CTH influences COSH yet does not determine it. The second part of this study will focus on simulations in microvascular networks from the rodent cerebral cortex. Actual estimates of COSH and CTH will then be given.

## Introduction

Microvessels are the primary site of gas exchange in the vertebrate microvascular system due to their large surface area. Energy metabolism is largely dependent on a continuous oxygen supply from the microcirculation which is actively regulated by the microvasculature. For instance, in the cerebral cortex, dilations of pial arteries (Chen et al., 2011) and penetrating arterioles (Tian et al., 2010) are essential components of neurovascular coupling. The smallest blood vessels are also involved in these processes. Capillary hyperemia was shown to occur prior to dilation of pial arteries (Hillman, 2014) and pericyte-mediated capillary dilations were observed to actively regulate cerebral blood flow (Hall et al., 2014). Therefore, an efficient and robust regulation of oxygen supply is highly dependent on the healthy function of microvessels.

Malfunctions in the microvasculature occur in many diseases and conditions. Cerebral small vessel disease plays a crucial role in stroke, dementia and aging (Pantoni, 2010). Cerebral pericytes, which were reported to regulate capillary diameter during functional activation (Hall et al., 2014), are susceptible to damage in ischemia (Yemisci et al., 2009) and to loss or degeneration in conditions such as aging, hypertension and diabetes (Østergaard et al., 2015). Exposure to β-amyloid is toxic to pericytes and amyloid accumulation often occurs in relation to Alzheimer's disease (Hamilton et al., 2010). These deposits characterize cerebral amyloid angiopathy which is associated with cerebral blood flow disturbance (Thal et al., 2009). Therefore, the study of these conditions requires a proper understanding of the consequences of capillary dysfunction on oxygen transport.

Capillary dysfunction can be quantified by capillary transit time heterogeneity (CTH) which is the standard deviation of the transit time distribution. In their seminal study, Jespersen and Østergaard (2012) showed using a theoretical model that the efficacy of oxygen extraction decreases with CTH. In particular, disturbed capillary flow patterns can decrease oxygen extraction even in the absence of changes in mean flow. CTH was linked to a number of diseases and conditions such as Alzheimer's disease (Østergaard et al., 2013a), stroke-like symptoms (Østergaard et al., 2013b), traumatic brain injury (Østergaard et al., 2014a) and ischemic heart disease (Ostergaard et al., 2014b).

In the study by Jespersen and Østergaard (2012), CTH was related to the oxygen extraction fraction by extending the Bohr-Kety-Crone-Renkin model which assumes homogeneous oxygen partial pressure (Po_2_) in the extravascular compartment. This flow diffusion equation relates the decrease in blood oxygen concentration to the capillary transit time and the oxygen partial pressure drop across the capillary wall by means of a single rate constant. This constant was fitted to yield a resting oxygen extraction fraction value of 0.3 and the tissue Po_2_ was required as a model input. Angleys et al. (2015) refined this model to determine tissue Po_2_ values that match metabolic oxygen consumption and the oxygen extraction fraction values predicted by the Bohr-Kety-Crone-Renkin model.

The distribution of Po_2_ levels at the distal ends of capillaries is a key determinant of tissue oxygenation. Even if blood flow to a given region is adequate to meet the oxygen needs of the tissue, a maldistribution of flow can lead to wide variations of end-capillary Po_2_. If the Po_2_ distribution is highly heterogeneous, low values in some capillaries can cause tissue hypoxia, whereas a uniform distribution of end-capillary Po_2_ tends to minimize tissue hypoxia, for a given overall oxygen supply. The heterogeneity of end-capillary Po_2_ can equivalently be expressed in terms of the heterogeneity of hemoglobin saturation (HS), which is functionally dependent on Po_2_ according to the oxy-hemoglobin saturation curve. This study therefore focuses on capillary outflow saturation heterogeneity (COSH), a measure of the variability of blood oxygen content at the distal end of capillaries where blood flows into venules. Elevated COSH may imply that a fraction of microvessels cannot supply oxygen to their surrounding tissue even if the average saturation is sufficiently high.

In previous modeling works (Jespersen and Østergaard, 2012; Angleys et al., 2015), the effects of CTH on brain oxygenation were quantified using blood oxygen levels. Both tissue Po_2_ and capillary HS were shown to depend on transit times through the erythrocyte velocity. Like other microvascular beds, the brain microvasculature has a heterogeneous geometric structure and hemodynamics, with substantial variability in path lengths, vessel diameters, flow rates, hematocrit, and tissue volumes supplied by individual capillaries. In parallel capillary arrays, the interaction among vessels reduces the heterogeneity of Po_2_ when RBC velocity and inlet oxygen concentration take different values in each capillary (Popel et al., 1986; Salathe, 2003). Besides, in the absence of CTH, irregular capillary spacings lead to a more heterogeneous oxygenation compared to regularly spaced arrays (Hoofd and Turek, 1996), which also affects the distribution of HS. Therefore, multiple factors beside CTH contribute to COSH, and CTH by itself does not provide a sufficient basis for understanding and predicting tissue oxygenation. The present study addresses the factors determining COSH and its relation to CTH, using theoretical models.

To compute COSH, models that describe the evolution of HSH in single and multiple capillaries are developed. Diffusive oxygen transfer among RBCs is shown to be the main physical mechanism for the reduction of HSH. The diffusive interaction between RBCs in single capillaries and between parallel capillaries is modeled based on ordinary differential equations. These interaction models are validated for a large range of physiological parameters using a computational model with individual moving RBCs (Lücker et al., 2014). Explicit formulas for the associated length and time scales are given and the resulting values are compared to RBC transit times and path lengths in capillaries. The modeling of COSH is an essential step toward an actual understanding of CTH and its relation to oxygen transport in MVNs. The resulting insights have potentially broad implications in the study of capillary dysfunction and related conditions.

## Materials and methods

Models for the diffusive interaction between RBCs in single capillaries and between multiple capillaries were developed based on ordinary differential equations that extend those used in Lücker et al. (2017). The interaction models were compared to our previously developed oxygen transport model with individual moving RBCs (Lücker et al., 2014).

Based on the observation that the RBC transit time is only one of multiple parameters that determine HS (Lücker et al., 2017), we asked the question: “Which phenomena cause a difference between CTH and COSH?” Two representative situations that contribute to a reduction of HSH were identified. The first one pertains to branchings where two capillaries with different HS levels are converging. This heterogeneity, which may result from different transit times, causes the rates of oxygen unloading from individual RBCs to differ. The second situation concerns parallel capillaries with different HS. In this case, the tissue volume supplied by each capillary can differ.

Diffusive interaction models are derived for these two representative situations. These simplified models directly highlight the variables that influence most the reduction of HSH. Then, the employed computational model (Lücker et al., 2014) is briefly outlined.

### Models for hemoglobin saturation based on ordinary differential equations

**Figure 1:**
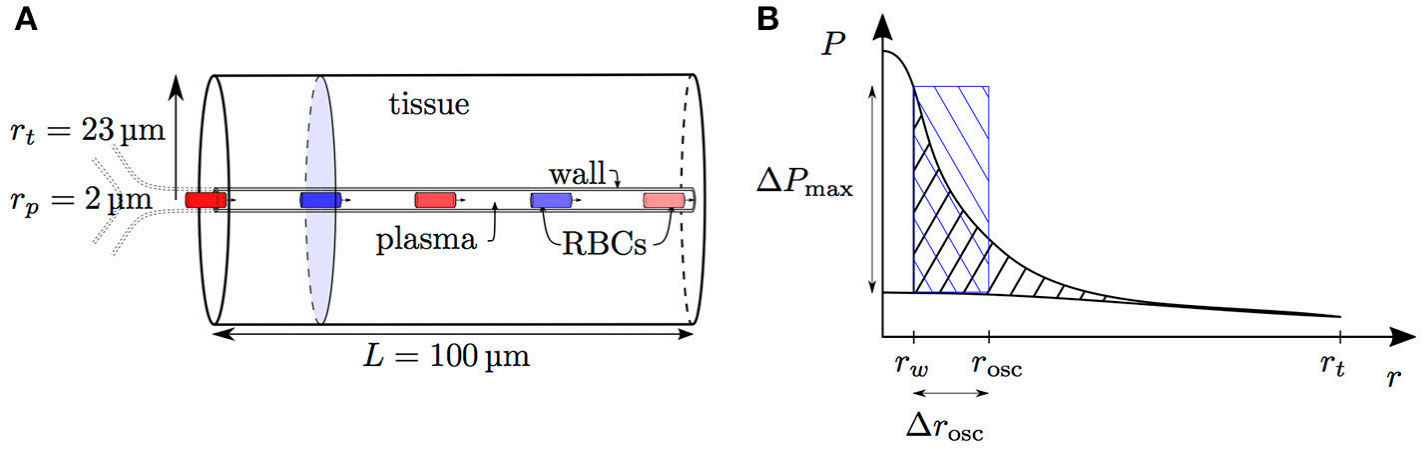
Schematics for RBC diffusive interaction. **(A)** Cylindrical domain used for the study of RBC diffusive interaction. In the modeled scenario, RBCs are flowing from a converging bifurcation into the domain with different HS values. The shaded tissue slice has area $\pi \left(r_{t}^{2}-r_{w}^{2}\right)$, where *r*_*t*_ is the tissue cylinder radius and *r*_*w*_ is the capillary endothelium radius. The term *j*_*t*_ defined in Equation (5) corresponds to the metabolic oxygen consumption in the tissue slice at a given axial position *x*. The plasma radius is denoted by *r*_*p*_ and the domain axial length by *L*. **(B)** Sketch for the definition of the integral spreading distance of Po_2_ oscillations (Equation 21). The top and bottom radial profiles indicate the maximal and minimal values of Po_2_ as RBCs are passing. The integral in cylindrical coordinates of the blue rectangle with width Δ*r*_osc_ is equal to that of the black hatched area.

The interaction models developed in this study are all formulated in an axisymmetric geometry with four distinct regions: RBCs, plasma, capillary endothelium and tissue, denoted by the indices *c*, *p*, *w*, and *t*, respectively. Cylindrical RBCs with radius *r*_*c*_ and volume *V*_rbc_ are employed. The domain geometry is described by its length *L*, the plasma radius *r*_*p*_, the outer capillary wall radius *r*_*w*_ and the tissue radius *r*_*t*_(*x*) which may be a function of the axial position *x* (Figure 1A). The arterial and venous capillary ends (also referred to proximal and distal ends) are denoted by the indices *a* and *v*.

In capillaries, RBCs flow in a single file with velocity *v*_rbc_ and the RBC linear density is defined to be the ratio of the length occupied by the RBCs to the total vessel length. It is related to the tube hematocrit *H*_*T*_ by

(1) $$\mu_{LD}=H_{T}{\left(\frac{r_{p}}{r_{c}}\right)}^{2}.$$

The equilibrium curve for hemoglobin and oxygen is modeled using the Hill equation

(2) $$S_{\text{eq}}(P)=\frac{P^{n}}{P^{n}+P_{50}^{n}},$$

where *n* is the Hill exponent and *P*_50_ the oxygen partial pressure at half-saturation. The inverse form of this equation

(3) $$P_{\text{eq}}(S)=P_{50}{\left(\frac{S}{1-S}\right)}^{1/n}$$

will often be required. Since the Hill equation is known to be inaccurate at low Po_2_ values, the more complex Adair equation (Popel, 1989) will also be used to estimate diffusive interaction between parallel capillaries.

The models developed here are based on the neglect of axial diffusion and the use of steady-state equations. These common assumptions (Hellums, 1977; Roy and Secomb, 2014; Lücker et al., 2017) reduce the evolution of HS to an ordinary differential equation. Based on the absence of axial diffusion, the oxygen outflux from the capillary at the axial position *x* is balanced by the metabolic oxygen consumption integrated over the tissue slice normal to the capillary at *x*, which is denoted by *j*_*t*_(*x*). The mass balance between the capillary and the tissue is given by

(4) $$\frac{\text{d}f(S)}{\text{d}x}=-j_{t}(x),$$

where *f*(*S*) is the convective oxygen flux through the capillary. Oxygen consumption is assumed to occur at a rate per unit volume *M*_0_ which is independent from tissue Po_2_. This simplification allows the existence of an analytical expression for Po_2_ in the tissue. Based on this assumption, the oxygen consumption in the tissue slice normal to *x* becomes

(5) $$j_{t}(x)=M_{0}\pi (r_{t}^{2}(x)-r_{w}^{2}),$$

as illustrated in Figure 1A.

Oxygen in the blood is present bound to hemoglobin in RBCs and dissolved in both plasma and RBCs. The total convective flux is given by

(6) $$f(S)=\;\nu_{\text{rbc}}(\pi r_{p}^{2}H_{D}C_{0}S+\pi r_{p}^{2}\alpha_{\text{eff}}P_{IV}),$$

where *C*_0_ = *N*_Hb_*V*_mol,O_2__ is the product of the heme concentration and the molar volume of oxygen, and *P*_*IV*_ is the intravascular Po_2_ which needs to be modeled. Here, the Fåhraeus effect is neglected, hence the discharge hematocrit *H*_*D*_ is set to *H*_*T*_. The effective oxygen solubility is defined by α_eff_ = *H*_*T*_α_*c*_ + (1 − *H*_*T*_)α_*p*_. The first term in the right-hand side of Equation (6) thus accounts for oxygen bound to hemoglobin and the second one represents dissolved oxygen in the capillary. The average RBC oxygen partial pressure *P*_*c*_ is assumed to be in equilibrium with HS, that is, *P*_*c*_ = *P*_eq_(*S*); in the plasma, the oxygen partial pressure is set to be constant and equal to *P*_*w*_, the oxygen partial pressure at the capillary outer wall. As in Lücker et al. (2017), we use the intravascular resistance coefficient *K*_*IV*_ defined by

(7) $$K_{IV}j_{t}=P_{c}-P_{w}.$$

Values of *K*_*IV*_ can be fitted from numerical simulations or obtained using an analytical formula (Lücker et al., 2017). This yields

(8) $$P_{IV}=H_{T}P_{\text{eq}}(S)+(1-H_{T})P_{w}=P_{\text{eq}}(S)-(1-H_{T})K_{IV}j_{t}.$$

We can now summarize the above equations to obtain the evolution equation for HS. For brevity, we define total oxygen convective capacity as

(9) $$Q_{{\text{O}}_{2}}(S)=\nu_{\text{rbc}}\left(\mu_{LD}\pi r_{c}^{2}C_{0}+\pi \;r_{p}^{2}\alpha_{\text{eff}}\frac{\text{d}P_{\text{eq}}}{\text{d}S}\right).$$

Equations (4), (6) and (8) result in

(10) $$Q_{{\text{O}}_{2}}(S)\frac{\text{d}S}{\text{d}x}=-j_{t}(x)+\nu_{\text{rbc}}\pi \;r_{p}^{2}\alpha_{\text{eff}}(1-H_{T})K_{IV}\frac{\text{d}j_{t}(x)}{\text{d}x}.$$

When the tissue domain is a straight cylinder, the last term vanishes. If the tissue radius is not constant, this term is nonzero but was found to be negligible. Therefore, it will be omitted in further derivations. However, this term was included in all numerical computations for completeness. Equation (10) can be recast in terms of the capillary transit time τ and integrated as

(11) $$S(\tau )=S_{a}-\int_{0}^{\tau }\frac{j_{t}(\nu_{\text{rbc}}t)}{\mu_{LD}\pi \;r_{c}^{2}C_{0}+\pi \;r_{p}^{2}\alpha_{\text{eff}}\frac{\text{d}P_{\text{eq}}}{\text{d}S}}$$

Thus, HS on the distal side is influenced by the RBC transit time, the oxygen consumption per unit length, hematocrit and vessel diameter. This description of distal blood oxygen concentration is more complete than the previously used Bohr-Kety-Crone-Renkin equation (Jespersen and Østergaard, 2012; Angleys et al., 2015). The BKCR model uses the normalized coordinate $\tilde{x}=x/L$ and reads

(12) $$\frac{\text{d}C}{\text{d}\tilde{x}}=-k\tau \left(\alpha_{\text{rbc}}P_{50}{\left(\frac{C}{B-C}\right)}^{1/n}-C_{t}\right),$$

where *C* is the bound oxygen concentration in RBCs, *B* is the maximal amount of oxygen bound to hemoglobin and *C*_*t*_ the oxygen concentration in the tissue. The model constant *k* was adjusted to obtain an oxygen extraction fraction of 0.3 in Jespersen and Østergaard (2012) and Angleys et al. (2015). In Equation (10), the counterpart of *k* is the inverse of *Q*_O_2__(*S*) (Equation 9) which is an explicit function of hematocrit and the vessel geometry.

Given the sink term *j*_*t*_(*x*), Equation (10) can be integrated numerically using a standard differential equation solver. The implementation in SciPy (Jones et al., 2001) of an explicit Runge-Kutta method of order 4(5) was used (Hairer et al., 1993).

In the modeling of diffusive integration between parallel capillaries, knowledge of the Po_2_ in the tissue will be required. This is achieved using the intravascular resistance coefficient and the Krogh model. From the HS *S* at an axial position *x*, the average RBC oxygen partial pressure *P*_*c*_ is obtained using Equation (3). The oxygen partial pressure *P*_*w*_ at the capillary outer wall is then given by Equation (7). Finally, the Krogh model for the oxygen partial pressure at a distance *r* from the capillary centerline reads

(13) $$P(x,r)=P_{w}(x)-\frac{M_{0}}{4D_{t}\alpha_{\text{t}}}\left[2r_{t}^{2}\ln\left(\frac{r}{r_{w}}\right)-r^{2}+r_{w}^{2}\right],\;r\geq r_{w}.$$

#### Diffusive interaction between RBCs in a single capillary

Capillary networks in the cerebral microvasculature form a mesh-like structure (Lorthois and Cassot, 2010; Blinder et al., 2013) with both diverging and converging bifurcations. At converging bifurcations, RBCs from either inflow branch may have different HS, for instance due to different transit times or hematocrit values. Here, we derive an interaction model for the evolution of HS saturation in a single capillary.

To describe this fluctuation, the HS is treated as a random variable *S*. The RBC interaction model is based on Fick's law as follows: the oxygen flux out of the capillary is assumed to be proportional to the oxygen partial pressure difference between the RBC and the tissue. In other words, we assume that

(14) $$Q_{{\text{O}}_{2}}(S)\frac{\text{d}S}{\text{d}x}=-C(P_{\text{eq}}(S)-P(r_{s})),$$

where *r*_*s*_ is a radial position which is independent from the fluctuations of *S* and where the Po_2_ fluctuations in the tissue are small (Figure 1B), and *C* is a proportionality factor that will be derived. From now on, averaged quantities will be denoted by an overline. In the next steps, the nonlinearity in *S* of the total convective oxygen capacity *Q*_O_2__(*S*) (Equation 9) will be ignored, which allows the simplification $\bar{Q_{{\text{O}}_{2}}\left(S\right)}=Q_{{\text{O}}_{2}}\left(\bar{S}\right)$. Based on this, the averaged mass balance is given by

(15) $$Q_{{\text{O}}_{2}}(\bar{S})\frac{\text{d}\bar{S}}{\text{d}x}=-j_{t}(x).$$

The averaging of Equation (14) combined with Equation (15) yields an expression for *C* which can be inserted into Equation (14). The terms can be rearranged as

(16) $$Q_{{\text{O}}_{2}}(S)\frac{\text{d}S}{\text{d}x}=-j_{t}(x)\left(1+\frac{P_{\text{eq}}(S)-\bar{P_{\text{eq}}(S)}}{\bar{P_{\text{eq}}(S)}-P(r_{s})}\right).$$

The oxygen partial pressure *P*(*r*_*s*_) still needs to be modeled. This is achieved by introducing the resistance coefficient for RBC diffusive interaction $K_{RI}=\left(\bar{P_{\text{eq}}\left(S\right)}-P\left(r_{s}\right)\right)/j_{t}\left(x\right)$, which is the only parameter of this model. Thus, the model equation for randomly distributed HS in a single capillary is given by

(17) $$Q_{{\text{O}}_{2}}(S)\frac{\text{d}S}{\text{d}x}=-j_{t}(x)-\frac{1}{K_{RI}}(P_{\text{eq}}(S)-\bar{P_{\text{eq}}(S)}).$$

By a suitable linearization, the nonlinear Equation (17) can be further simplified to an evolution equation for the standard deviation of *S*. Here, we again assume that the function *P*_eq_ (Equation 3) is linear around $\bar{S}$ and that the derivative $\frac{\text{d}}{\text{d}x}\frac{\text{d}P_{\text{eq}}}{\text{d}S}\left(\bar{S}\right)$ can be neglected. It follows that

(18) $$Q_{{\text{O}}_{2}}(\bar{S})\frac{\text{d}}{\text{d}x}(S-\bar{S})=-\frac{1}{K_{RI}}{\frac{\text{d}P_{\text{eq}}}{\text{d}S}|}_{\bar{S}}(S-\bar{S}).$$

Using the above assumptions, the standard deviation of HS σ_*S*_ satisfies the differential equation

(19) $$Q_{{\text{O}}_{2}}(\bar{S})\frac{\text{d}\sigma_{S}}{\text{d}x}=-\frac{\sigma_{S}}{K_{RI}}{\frac{\text{d}P_{\text{eq}}}{\text{d}S}|}_{\bar{S}}.$$

This equation can be solved numerically given the average HS $\bar{S}$ which is itself obtained by integrating Equation (10).

The resistance coefficient *K*_*RI*_ describes the resistance to the Po_2_ drop between the RBC and a location in the tissue where Po_2_ oscillations are small. Since oscillations resulting from fluctuating capillary Po_2_ decay exponentially with distance into the tissue, there is no precise definition of this location and no exact formula for *K*_*RI*_ can be derived. However, this coefficient can be fitted based on numerical simulations and compared to a measure for the spreading distance of Po_2_ oscillations into the tissue. First, *K*_*RI*_ is decomposed as *K*_*RI*_ = *K*_*IV*_ + *K*_*OS*_, where *K*_*IV*_ is the intravascular resistance coefficient (Lücker et al., 2017) and *K*_*OS*_ represents the extravascular contribution to *K*_*RI*_. Second, we define a characteristic penetration radius *r*_osc_ for Po_2_ oscillations by

(20) $$\pi (r_{\text{osc}}^{2}-r_{w}^{2})\Delta P_{\text{max}}=\int_{r_{w}}^{r_{t}}r\Delta P(r)\text{d}r,$$

where Δ*P* is the radially varying fluctuation in tissue Po_2_ (Figure 1B). Finally, the integral oscillation spreading distance Δ*r*_osc_ is defined as

(21) $$\Delta r_{\text{osc}}=r_{\text{osc}}-r_{w}.$$

This quantity can be obtained from the results of the computational model. We will show that Δ*r*_osc_ is an accurate predictor for the model coefficient *K*_*OS*_.

#### Diffusive interaction between parallel capillaries

**Figure 2:**
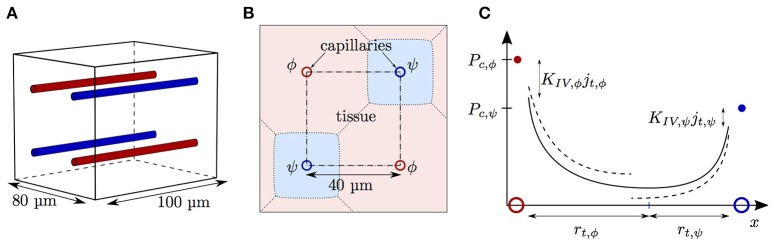
Schematics for capillary diffusive interaction. **(A)** Sketch of the geometry with four parallel capillaries. **(B)** Transverse view of the computational domain with four parallel capillaries. The shaded areas represent the tissue regions supplied by each capillary. Solid lines: boundary of the representative domain with periodic boundary condition; dash-dotted lines: actual computational domain with symmetry boundary condition; dotted lines: boundary of the tissue region supplied by each capillary. **(C)** Sketch of the tissue Po_2_ between two capillaries with different RBC Po_2_ values *P*_*c*,ϕ_ (red dot) and *P*_*c*,ψ_ (blue dot). The colored circles indicate the locations of the capillaries ϕ and ψ. Solid line: continuous Po_2_ profile with adapted tissue radii *r*_*t*,ϕ_ and *r*_*t*,ψ_; dashed line: discontinuous Po_2_ profile under the assumption of equal oxygen fluxes out of the capillaries.

Having examined the diffusive interaction between heterogeneously saturated RBCs in the same capillary, we now consider the diffusive interaction between capillaries with different saturation levels. For our analysis, four parallel capillaries with concurrent blood flow are considered where both pairs of diagonally opposed capillaries will be denoted by the indices ϕ and ψ, respectively (Figure 2).

Given different HS values *S*_ϕ,*a*_, *S*_ψ,*a*_ at the proximal inlets, we aim to derive the evolution of *S* in both capillaries. To do this, the tissue region supplied by each capillary is approximated by a cylinder with varying radius which will be determined using the continuity of tissue Po_2_. As above, the neglect of axial diffusion allows tissue slices that are orthogonal to the capillary to be decoupled. Let *A* be the area of the normal domain slice supplied by the two model capillaries ϕ and ψ. Mass conservation implies that the oxygen flux at *x* out of both model capillaries balances the metabolic oxygen consumption in the tissue slice normal to *x*:

(22) $$j_{t,\phi }(x)+j_{t,\psi }(x)=M_{0}(A-2\pi r_{w}^{2}).$$

Using the intravascular resistance coefficient and the Krogh model (Equation 13), the continuity of tissue Po_2_ at the interface between the Krogh cylinders is given by

(23) $$\begin{array}{l} P_{c,\phi }(x)-K_{IV,\phi }j_{t,\phi }(x)-\Delta P_{EV}(j_{t,\phi }(x))=P_{c,\psi }(x)-K_{IV,\psi }j_{t,\psi }(x)\\ \;-\Delta P_{EV}(j_{t,\psi }(x)), \end{array}$$

where Δ*P*_*EV*_(*j*_*t*_) is the extravascular Po_2_ drop associated to the local oxygen outflux *j*_*t*_. Based on the right-hand side of Equation (13), it is given by

(24) $$\begin{array}{l} \Delta P_{EV}(j_{t})=\{\begin{array}{ll} \frac{M_{0}}{4D_{t}\alpha_{\text{t}}}\left[2r_{t}^{2}\ln\left(\frac{r_{t}}{r_{w}}\right)-r_{t}^{2}+r_{w}^{2}\right], & j_{t}\geq 0,\\ 0,\; & j_{t}<0. \end{array}\\ \;r_{t}=\sqrt{\frac{j_{t}}{M_{0}\pi }+r_{w}^{2}} \end{array}$$

Given *P*_*c*,ϕ_ and *P*_*c*,ψ_, the nonlinear equation system formed by Equation (22) and (23) can be solved numerically for *j*_*t*, ϕ_ and *j*_*t*,ψ_. Then, Equation (10) is solved in both model capillaries one step forward using an explicit differential equation integrator. This model will be referred to as nonlinear Krogh-based model and can be applied to capillaries with different flows and radii. Cases where one capillary is supplied with oxygen by the other (for instance, *j*_*t*,ψ_ < 0) are captured by this formulation.

A slight simplification in Equation (23) provides an explicit expression for the oxygen flux out of both model capillaries. Under the assumption that both capillaries have the same geometry and linear density, the intravascular resistance coefficient *K*_*IV*_ takes the same value in both capillaries, so Equation (23) can be rearranged as

(25) $$\begin{array}{l} \frac{P_{c,\phi }(x)-P_{c,\psi }(x)}{M_{0}}=\left(K_{IV}\pi -\frac{1}{4D_{t}\alpha_{t}}\right)(r_{t,\phi }^{2}-r_{t,\psi }^{2}) \end{array}$$

(26) $$\begin{array}{l} \;+\frac{1}{2D_{t}\alpha_{t}}\left(r_{t,\phi }^{2}\ln\left(\frac{r_{t,\phi }}{r_{w}}\right)-r_{t,\psi }^{2}\ln\left(\frac{r_{t,\psi }}{r_{w}}\right)\right). \end{array}$$

The assumption that

(27) $$\ln\left(\frac{r_{t,\phi }}{r_{w}}\right)≃\ln\left(\frac{r_{t,\psi }}{r_{w}}\right)≃\ln\left(\frac{r_{t,\text{mean}}}{r_{w}}\right),$$

where $r_{t,\text{mean}}=\sqrt{\frac{1}{2}\left(r_{t,\phi }^{2}+r_{t,\psi }^{2}\right)}$, yields an explicit expression for the oxygen outflux

(28) $$j_{t,\phi }(x)=M_{0}\pi (r_{t,\text{mean}}^{2}-r_{w}^{2})+\frac{P_{c,\phi }(x)-P_{c,\psi }(x)}{2K_{IV}+\frac{1}{\pi D_{t}\alpha_{\text{t}}}\left(\ln\left(\frac{r_{t,\text{mean}}}{r_{w}}\right)-\frac{1}{2}\right)}$$

and a similar expression for *j*_*t*,ψ_(*x*). We now define the resistance coefficient *K*_*CI*_ for diffusive interaction between capillaries as

(29) $$K_{CI}=K_{IV}+\frac{1}{2\pi D_{t}\alpha_{\text{t}}}\left(\ln\left(\frac{r_{t,\text{mean}}}{r_{w}}\right)-\frac{1}{2}\right).$$

By using the average oxygen outflux $j_{t}=M_{0}\pi \left(r_{t,\text{mean}}^{2}-r_{w}^{2}\right)$, the evolution equations for HS in both model capillaries become

(30) $$Q_{{\text{O}}_{2},\phi }(S_{\phi })\frac{\text{d}S_{\phi }}{\text{d}x}=-j_{t}-\frac{1}{2K_{CI}}(P_{c,\phi }(x)-P_{c,\psi }(x))$$

(31) $$Q_{{\text{O}}_{2},\psi }(S_{\psi })\;\frac{\text{d}S_{\psi }}{\text{d}x}=-j_{t}-\frac{1}{2K_{CI}}(P_{c,\psi }(x)-P_{c,\phi }(x)).$$

This model will be referred to as explicit Krogh-based model. To derive this equation, the respective linear densities in both capillaries were assumed to be equal. However, the respective RBC velocities were still allowed to be different. Under the assumption that *v*_rbc_ is equal in both capillaries, the linearization of *P*_eq_ around the average HS $\bar{S}=\frac{1}{2}\left(S_{\phi }+S_{\psi }\right)$ yields the following evolution equation for the saturation difference Δ*S* = *S*_ϕ_ − *S*_ψ_ between both model capillaries:

(32) $$Q_{{\text{O}}_{2}}(\bar{S})\frac{\text{d}\Delta S}{\text{d}x}=-\frac{\Delta S}{K_{CI}}{\frac{\text{d}P_{\text{eq}}}{\text{d}S}|}_{\bar{S}}.$$

This third model will be referred to as linearized capillary interaction model. This equation leads to the definition of the characteristic length scale *L*_*CI*_ for diffusive interaction between parallel capillaries

(33) $$L_{CI}=K_{CI}Q_{{\text{O}}_{2}}(\bar{S}){\left({\frac{\text{d}P_{\text{eq}}}{\text{d}S}|}_{\bar{S}}\right)}^{-1}.$$

Similarly, the characteristic time scale τ_*CI*_ is defined as

(34) $$\tau_{CI}=\frac{L_{CI}}{\nu_{\text{rbc}}}=K_{CI}\left(\mu_{LD}\pi \;r_{c}^{2}C_{0}{\left({\frac{\text{d}P_{\text{eq}}}{\text{d}S}|}_{\bar{S}}\right)}^{-1}+\pi \;r_{p}^{2}\alpha_{\text{eff}}\right).$$

Thus it is independent from the RBC velocity and depends on linear density, the average HS $\bar{S}$ and the geometry. These characteristic quantities will be compared to fits obtained using the computational model presented below.

### Computational model

The results of the models for RBC and capillary diffusive interactions were compared with numerical solutions to the advection-diffusion-reaction equations for oxygen and hemoglobin. The reaction rates between both quantities are coupled based on Clark et al. (1985) with

(35) $$f(P,S)=\{\begin{array}{ll} k_{-}\left(S-(1-S){\left(\frac{P}{P_{50}}\right)}^{n}\right)\; & \text{inside RBCs,}\\ 0 & \text{outside RBCs,} \end{array}$$

where *k*_−_ is the reaction rate. Metabolic oxygen consumption was modeled using zero-th order kinetics as

(36) $$M(P)=\{\begin{array}{cc} M_{0} & \text{inside tissue},\\ 0 & \text{outside tissue}. \end{array}$$

This was chosen instead of the commonly used Michaelis-Menten kinetics to facilitate the comparison between the interaction models and the computational model. The oxygen transport equation is given by

(37) $$\frac{\partial \alpha P}{\partial t}+v\;\cdot \;\nabla (\alpha P)=\nabla \;\cdot \;(D\alpha \nabla P)+C_{0}f(P,S)-M(P),$$

where ***v*** is the plasma velocity. In RBCs, the evolution of HS follows

(38) $$\frac{\partial S}{\partial t}+v\;\cdot \;\nabla S=\nabla \;\cdot \;(D_{\text{Hb}}\nabla S)-f(P,S),$$

where *D*_Hb_ is the diffusion coefficient of hemoglobin in RBCs.

For simulations in a single capillary or parallel capillaries, these equations were solved using the finite-volume method with moving RBCs (Lücker et al., 2014).

### Model parameters

The heterogeneity of HS was investigated in different computational domains. The physiological parameters were chosen to match the mouse cerebral cortex. The diffusive interaction between RBCs was studied in a two-dimensional cylindrical domain with radius *r*_*t*_ = 23 μm, which corresponds to the distances between nuclei of neurons and capillaries (Tsai et al., 2009). Unless stated otherwise, a domain length *L* = 100 μm was chosen. This length is smaller than the average capillary path length of 343 μm measured by Sakadžić et al. (2014). The domain length influence will be addressed below. Cylindrical RBCs with volume *V*_rbc_ = 59 μm^3^ and radius *r*_*c*_ = 1.5 μm were employed. The capillary lumen diameter was set to *r*_*p*_ = 2.0 μm, which is typical in the rodent cerebral cortex (Tsai et al., 2009), and the endothelium thickness to 0.6 μm (Bertossi et al., 1997), so that the endothelium radius was *r*_*w*_ = 2.6 μm. At the tissue boundary, the gradient of the Po_2_ field was set to zero. In this domain, the grid cell size was set to Δ*x* = Δ*y* = 0.3 μm in the capillary. The radial grid spacing in the tissue was smoothly increased to save computational effort, so that Δ*y* was four times higher at the tissue boundary than in the capillary. The grid spacing in the RBC meshes was set to Δ*x*_rbc_ = Δ*y*_rbc_ = 0.1 μm. The time step size was set to Δ*t* = Δ*x*/*v*_rbc_. All simulations were ran until a statistical steady state was reached.

The diffusive interaction between capillaries was investigated in an array with four parallel capillaries with radius *r*_*p*_ = 2.0 μm. The symmetry of the domain allowed that only one quarter of each capillary had to be simulated (Figure 2B). The normal Po_2_ gradient was set to zero at each boundary plane. A spacing of 40 μm between the capillaries was chosen, which yields an averaged supplied tissue volume per capillary very close to that of a cylinder with radius *r*_*t*_ = 23 μm. In these simulations, the RBC radius was set to *r*_*c*_ = 1.6 μm and the endothelium radius to *r*_*w*_ = 2.5 μm. For this three-dimensional domain, a coarser grid spacing than in the two-dimensional cylinder was chosen. The grid cell size in the tissue away from the capillaries was set to 1 μm. At ≤ 8 μm from the capillaries, the grid was refined by a factor two to better resolve the high oxygen gradients in and close to the capillaries. The grid cell size in the RBCs was set to Δ*x*_rbc_ = 0.25 μm and the time step to Δ*t* = Δ*x*/*v*_rbc_. Since the HS difference Δ*S*_*v*_ between the venous ends of the capillaries is our main quantity of interest here, the grid spacing needs to be sufficiently high to accurately resolve this quantity. A grid convergence study showed that doubling the spatial resolution in each dimension and reducing the time step correspondingly increases Δ*S*_*v*_ by < 2.2%. Therefore, all simulations were run with the grid resolution described above, since it provides a good compromise between accuracy and run time (~20 h per simulation on a single core). The coarser grid resolution in the tissue was found not to affect the values of Δ*S*.

The metabolic rate of oxygen consumption was set to 10^−3^ μm^3^ O_2_ μm^−3^ s^−1^, which is within the range of values measured in the anesthetized rodent cerebral cortex (Zhu et al., 2013), using a brain density of 1.05 g cm^−3^ and the ideal gas law at body temperature for the molar volume of oxygen (2.544 × 10^4^ ml O_2_/(mol O_2_)). The intravascular resistance coefficient, which is used for the model coefficients *K*_*RI*_ and *K*_*CI*_ (Equation 29), was determined using the formula *K*_*IV*_ = 0.5*K*_*IV*, 0.5_/μ_*LD*_ (Lücker et al., 2017), with the difference that convective transport of dissolved oxygen content was included in Equation (10). For *r*_*c*_ = 1.5 μm, the value of *K*_*IV*, 0.5_ was 5.15 mmHg μm s/(μm^3^ O_2_). In parameter studies, the RBC velocity will be varied between 0.4 and 2.0 mm/s and the linear density between 0.2 and 0.6. When these parameters are fixed, *v*_rbc_ will be set to 1.0 mm/s and μ_*LD*_ to 0.3. For *r*_*c*_ = 1.5 μm, this yields a RBC flow equal to *v*_rbc_μ_*LD*_/*L*_rbc_ = 40.7 cells/s. These values are typical for the rodent brain (Parpaleix et al., 2013; Lyons et al., 2016). The other physiological parameters are given in Table 1.

**Table 1:** Parameter values.

**Parameter**	**Description**	**Value**	**Units**	**References**
α_rbc_	O_2_ solubility in RBCs	3.38 × 10^−5^	ml O_2_ mmHg^−1^ cm^−3^	Altman and Dittmer, 1971
α_p_	O_2_ solubility in the plasma	2.82 × 10^−5^	ml O_2_ mmHg^−1^ cm^−3^	Christoforides et al., 1969
α_w_	O_2_ solubility in the capillary wall	3.89 × 10^−5^	ml O_2_ mmHg^−1^ cm^−3^	α_t_
α_t_	O_2_ solubility in the tissue	3.89 × 10^−5^	ml O_2_ mmHg^−1^ cm^−3^	Mahler et al., 1985
*D*_rbc_	O_2_ diffusivity in RBCs	9.5 × 10^−6^	cm^2^ s^−1^	Clark et al., 1985
*D*_p_	O_2_ diffusivity in the plasma	2.18 × 10^−5^	cm^2^ s^−1^	Goldstick et al., 1976
*D*_w_	O_2_ diffusivity in the capillary wall	8.73 × 10^−6^	cm^2^ s^−1^	Liu et al., 1994
*D*_t_	O_2_ diffusivity in the tissue	2.41 × 10^−5^	cm^2^ s^−1^	Bentley et al., 1993
*D*_Hb_	Hemoglobin diffusivity in RBCs	1.44 × 10^−7^	cm^2^ s^−1^	Clark et al., 1985
*k*_−_	Dissociation rate constant	44	s^−1^	Clark et al., 1985
*n*	Hill exponent	2.64	–	Fitted from Watanabe et al. (2008)
*N*_Hb_	Total heme density	2.03 × 10^−5^	mol cm^−3^	Clark et al., 1985
*P*_50_	Po_2_ at hemoglobin half-saturation	47.9	mmHg	Fitted from Watanabe et al. (2008)
*r*_p_	Radius of capillary lumen	2.0	μm	Tsai et al., 2009
*V*_mol,_O__2__	O_2_ molar volume at 36.9°C	2.54 × 10^4^	ml O_2_ mol^−1^	Ideal gas law
*V*_rbc_	RBC volume	59.0	μ*m*^3^	Shirasawa, 2003

Equations (37) and (38) were solved using a custom written extension of the open-source computational fluid dynamics library OpenFOAM 2.3.0 (Weller et al., 1998). The equations were discretized as explained in Lücker et al. (2014).

## Results

The evolution of HSH was simulated in the geometries shown in Figure 1A, 2A, and compared to the RBC and capillary interaction models.

### Diffusive interaction between RBCs

**Figure 3:**
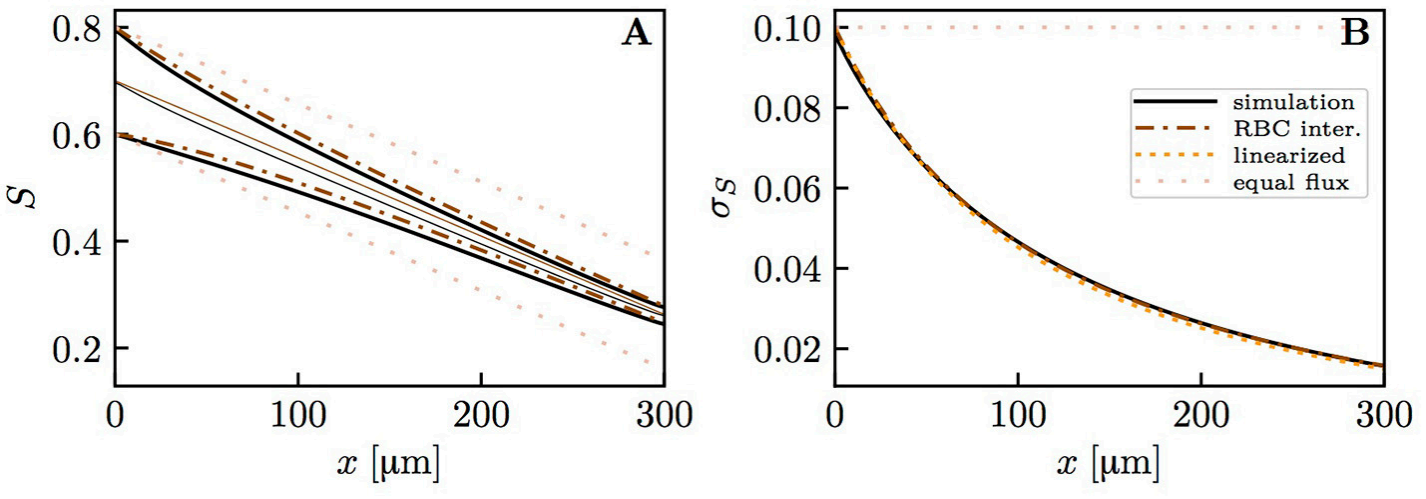
Hemoglobin saturation profiles with alternating inlet values in the cylindrical geometry. μ_*LD*_ = 0.3; *v*_rbc_ = 1.0 mm/s. Solid lines: numerical simulation; dash-dotted lines: RBC interaction model; dotted line: linearized RBC interaction model; pale dotted lines: assumption of equal oxygen flux out of the RBCs. **(A)** HS profile of heterogeneously saturated RBCs (thin line: average). **(B)** Standard deviation profile of *S*.

The diffusive interaction between RBCs with different HS was investigated in a cylindrical tissue domain (Figure 1A). This single-capillary setup with differently saturated RBCs aims to represent a capillary after a converging bifurcation where RBCs with different transit times are flowing in. The simplest model for the inlet HS of RBCs is when erythrocytes alternatingly take two fixed saturation values (one value per upstream branch). Figure 3 shows the evolution of HS in a capillary with length *L* = 300 μm with inlet values *S* = 0.8 and 0.6. The standard deviation σ_*S,v*_ of the HS from the numerical model at the venous end is approximately seven times lower than at the inlet. The values of σ_*S*_ from the RBC interaction model is almost indistinguishable from the numerical results (Figure 3B) when the model coefficient *K*_*RI*_ is fitted to match the standard deviation from the computational model (here, *K*_*RI*_ = 11.1 mmHg μm s/(μm^3^ O_2_)). The coefficient was fitted to minimize the model error $\int_{0}^{L}||\sigma_{S,\text{model}}\left(x\right)-\sigma_{S,\text{simul}}\left(x\right)|{|}_{2}^{2}\text{d}x$. The linearized RBC diffusive interaction model with the same value of *K*_*RI*_ also agrees very well with the numerical results, although it very slightly underestimates σ_*S*_. The simulated values of σ_*S*_ were also fitted with a single exponential function of the form *f*(*x*) = *a* exp(*x*/*L*_*RI*_). Since this fit is also very good, our results can be expressed in terms of the characteristic decay length *L*_*RI*_ and the related decay time τ_*RI*_ = *L*_*RI*_/*v*_rbc_. These first results suggest that HSH can be considerably reduced by diffusive interaction between RBCs within a single capillary.

To reduce the computational effort in further parameter studies, we compared the results obtained with domain lengths of 100 μm (*S*_*a*_ = 0.6 and 0.4) and 300 μm (*S*_*a*_ = 0.8 and 0.6, respectively). The fitted value of *K*_*RI*_ in the short domain was only 4.6% lower than in the long domain. Therefore, the domain length does not have a major influence on the results and from now on we will use *L* = 100 μm. In the mouse cerebral cortex, an average capillary path length of 343 μm was measured by Sakadžić et al. (2014). The similar values of *K*_*RI*_ with *L* = 100 μm and 300 μm show that it is not necessary to simulate whole capillary paths to estimate the model coefficient *K*_*RI*_, which in turn determines *L*_*RI*_ and τ_*RI*_. Additionally, a uniform random distribution of HS at the inflow of a 2 × 2 parallel capillary array yields very similar results (Figure S1). To investigate the dependence on the considered organ, a simulation was run with parameters for the working hamster retractor muscle as in Eggleton et al. (2000). The resulting evolution of HSH is qualitatively the same as with physiological parameters for the mouse cerebral cortex (Figure S2). This shows model robustness with respect to the inflow value of *S*, the boundary condition for tissue Po_2_ and the considered organ.

**Figure 4:**
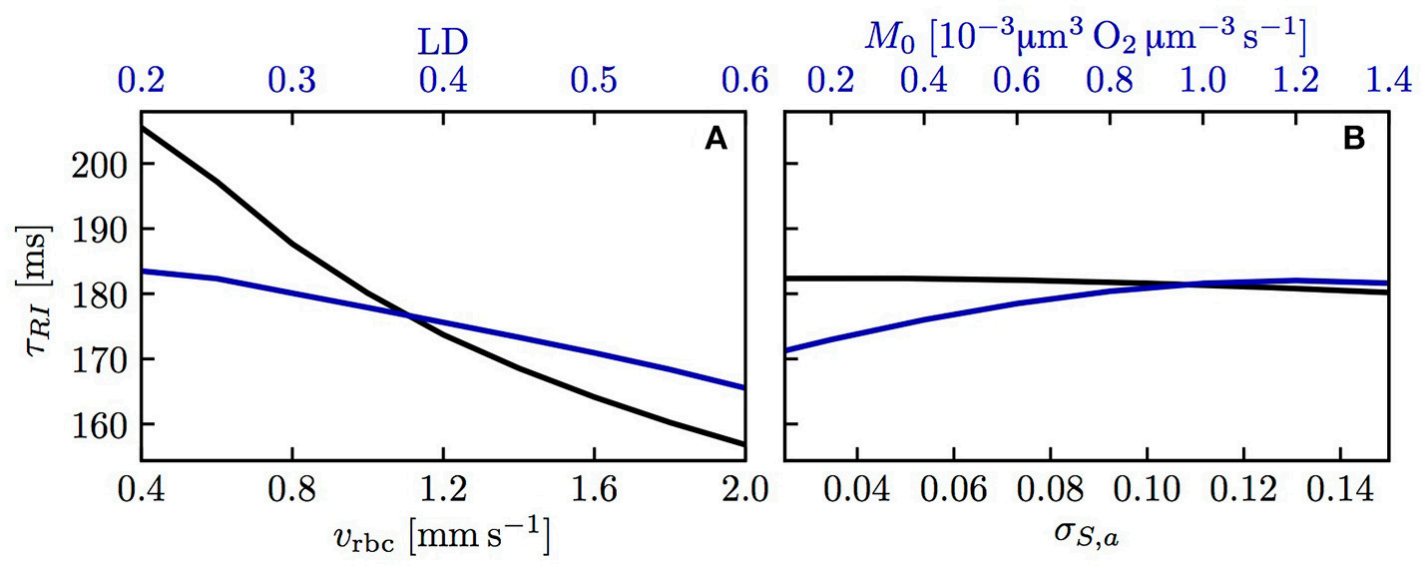
Decay time scale τ_*RI*_ for RBC diffusive interaction. The time scale τ_*RI*_ was obtained with an exponential fit of the standard deviation of HS from simulations across a range of parameters. **(A)** Linear density and RBC velocity; **(B)** oxygen consumption rate and standard deviation of HS at the inlet.

We now examine the influence of model parameters such as linear density, RBC velocity, oxygen consumption rate and HS difference at the inlet on the results. Figure S3 shows that the RBC interaction models with fitted *K*_*RI*_ perform very well across a wide range of parameters. The relative model error in σ_*S,v*_ normalized by the standard deviation drop from the numerical model σ_*S,a*_ − σ_*S,v*_ is ≤ 2% for the initial model and ≤ 4% for the linearized model across the whole parameter range. Additionally, the exponential fit to the numerical results also matches very well the numerical results (< 2% error), which confirms that the decay length *L*_*RI*_ and decay time τ_*RI*_ introduced above can used to compare results. The decay time τ_*RI*_ decreases from 206 to 157 ms when *v*_rbc_ increases from 0.4 to 2.0 mm/s, but is rather insensitive to the linear density (10.2% decrease when μ_*LD*_ increases from 0.2 to 0.6, Figure 4). The oxygen consumption rate has an even smaller influence on τ_*RI*_ (6.3% variation), while the inlet standard deviation of HS almost does not affect it (1.2% variation).

**Figure 5:**
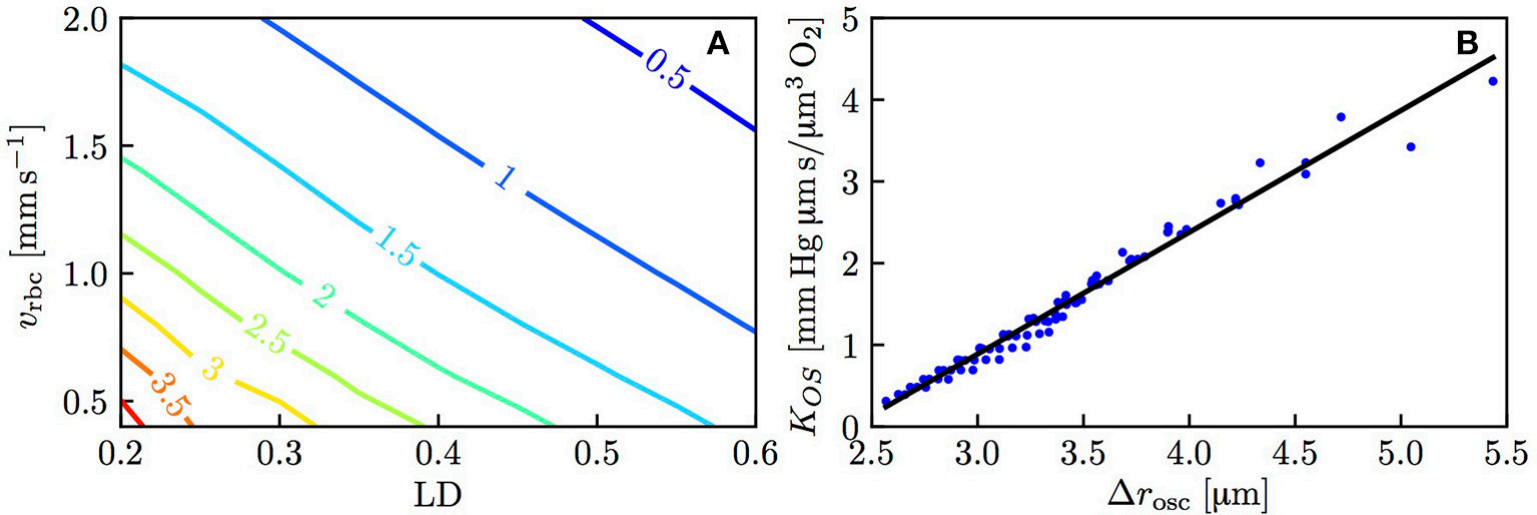
Model coefficient *K*_*OS*_ for RBC diffusive interaction. **(A)** *K*_*OS*_ in mmHg μm s/(μm^3^ O_2_) as a function of linear density and RBC velocity. **(B)** *K*_*OS*_ as a function of the integral spreading distance of Po_2_ oscillations defined in Equation (21); solid line: linear fit with slope 1.49 and intercept −3.58.

The above results show that the RBC interaction models agree closely with numerical simulations when using fitted values of *K*_*RI*_. To show the models' predictive power, it is necessary to characterize this model coefficient which was decomposed as *K*_*IV*_ + *K*_*OS*_, where *K*_*OS*_ was related to the spreading distance of Po_2_ oscillations in the tissue due to individual passing erythrocytes. Figure 5A shows the dependence of *K*_*OS*_ on linear density and RBC velocity. The plot of Δ*r*_osc_ against *K*_*OS*_ for all the simulated values of linear density and RBC velocity (Figure 5B) shows a strong correlation between these two quantities (Pearson's correlation coefficient *r* = 0.989). Therefore, consistently with our initial assumption (Equation 14), the model coefficient for RBC diffusive interaction is closely related to the Po_2_ oscillations in the tissue.

### Diffusive interaction between parallel capillaries

The capillary diffusive interaction models are now compared to our computational model for oxygen transport. Numerical simulations were run in an array of four straight, parallel capillaries (Figure 2A). In the two pairs of diagonally opposed capillaries, two different inlet values of HS *S*_*a*,ϕ_, *S*_*a*,ψ_ were chosen. The evolution of *S*_ϕ_, *S*_ψ_ and the HS difference Δ*S* = |*S*_ϕ_ − *S*_ψ_| were computed with the numerical model and compared to predictions from the three interaction models for the oxygen flux out of the capillaries (nonlinear Krogh-based model, explicit model and linearized model for Δ*S*). Additionally, theoretical results based on the assumption of equal oxygen outflux will be shown to highlight the effects of capillary diffusive interaction. Unless otherwise stated, the average value of *S*_*a*_ over all capillaries was 0.7 and the capillaries were 40 μm apart. The linear density and the erythrocyte velocity were set to 0.3 and 1.0 mm/s, respectively.

**Figure 6:**
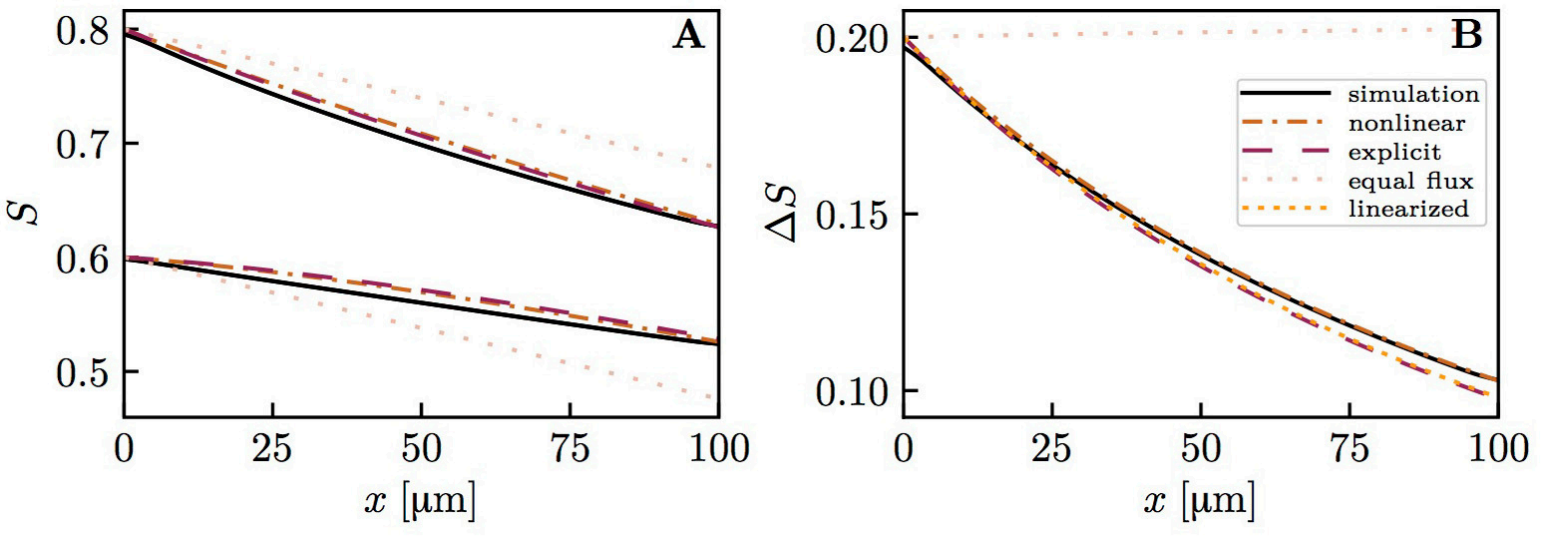
Hemoglobin saturation profiles in parallel capillaries. μ_*LD*_ = 0.3; *v*_rbc_ = 1.0 mm/s. Solid lines: numerical simulation; dash-dotted lines: nonlinear Krogh-based model; dashed lines: explicit Krogh-based model; dotted line: linearized model for Δ*S*; pale dotted lines: equal outflux assumption. **(A)** HS profile in both model capillaries. **(B)** HS difference between both capillaries.

Figure 6 shows HS profiles along both capillary pairs from the numerical model and the interaction models. The mean HS from the models matches very well the simulated results, which shows that mass conservation is fulfilled (the nonlinear and explicit Krogh-based models yield the same mean *S*, hence only the former is shown). In this setup, the HS difference between both capillary pairs drops by ~ 50% over 100 μm. This decrease is captured well by each interaction model, albeit slightly underestimated by the explicit Krogh-based model and the linearized model. Figure 6 also illustrates that the assumption of equal oxygen outfluxes cannot be used in the present context. As above, the underestimation of *S* by the interaction models away from the domain ends is caused by the absence of axial diffusion (Lücker et al., 2017). These first results suggest a strong reduction of HSH between parallel capillaries.

To show model robustness, several input parameters were varied and the predicted drop in HS difference Δ*S*_*a*_ − Δ*S*_*v*_ was compared to numerical simulation results. The spacing between capillaries, the oxygen consumption rate, the RBC velocity and linear density were investigated (Figure S4). In almost all cases, the nonlinear Krogh-based model shows the best agreement with numerical results (relative error ≤ 4% except at very low oxygen consumption rates). The explicit Krogh-based model and the linearized model perform almost equally well, with relative errors ≤ 8%. Similarly, simulations with physiological parameters for the working hamster retractor muscle yield results that are very similar to those with parameters for the mouse cerebral cortex (Figure S5). These parameter studies show that capillary diffusive interaction models perform well over a large range of physiological parameters.

**Figure 7:**
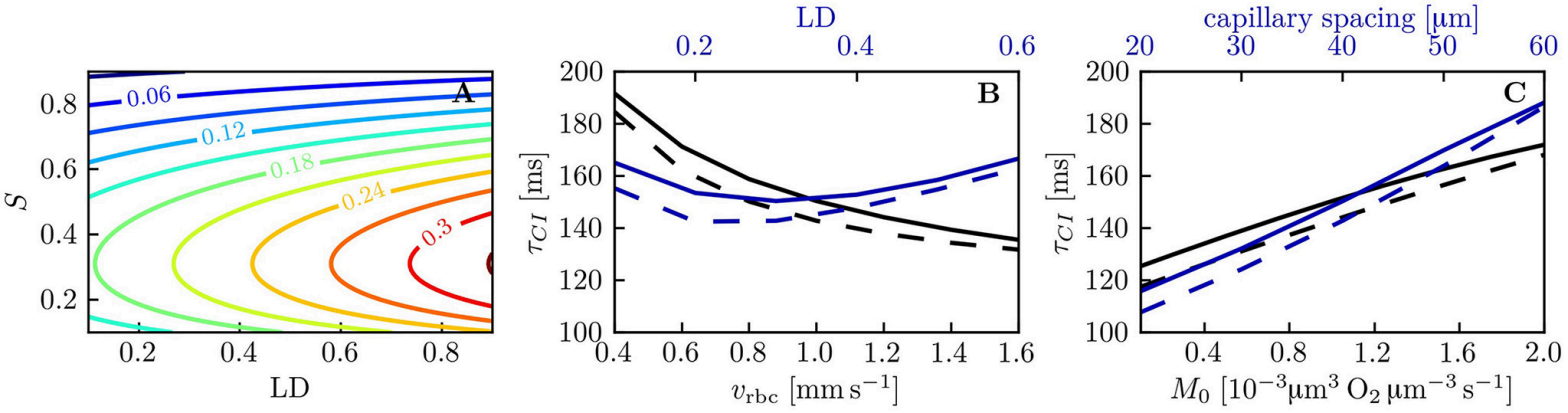
Decay time scale τ_*CI*_ for capillary diffusive interaction. **(A)** Theoretical decay time scale for capillary diffusive interaction. The contour values were obtained with Equation (34) for a capillary spacing of 40 μm (*r*_*t*,mean_ = 22.6 μm, *r*_*c*_ = 1.6 μm, *r*_*p*_ = 2.0 μm, *r*_*w*_ = 2.5 μm). **(B,C)** Decay time scale τ_*CI*_ of the HS difference between parallel capillaries across a range of parameters: linear density, RBC velocity **(B)**, capillary spacing and metabolic consumption rate of oxygen **(C)**. Solid lines: exponential fit to the simulated Δ*S*; dashed lines: theoretical value obtained with Equation (34) and the same parameters as in **(A)**.

The capillary interaction models rely on a single model parameter *K*_*CI*_ defined in Equation (29). Unlike the coefficient *K*_*RI*_ for RBC diffusive interaction, the expression for *K*_*CI*_ only depends on the intravascular resistance coefficient *K*_*IV*_ which can be determined based on numerical simulations (Lücker et al., 2017). Therefore, given a suitable value of *K*_*IV*_ and mean HS $\bar{S}$, the decay length and time scales *L*_*CI*_ and τ_*CI*_ (Equations (33) and (34), respectively) can be computed analytically. Figure 7A shows values of τ_*CI*_ for a range of linear densities and mean HS values. The decay time scale increases with linear density and attains its highest values at *S* ≃ 0.3, where d*P*_eq_/d*S* attains its minimum with the employed parameters for the Hill equation (Equation 2). Similarly to RBC diffusive interaction, the simulated values of Δ*S* are very well fitted by exponential decays. Figure 7B,C show a comparison of the analytical and the fitted decay time scale τ_*CI*_ for different values of capillary spacing, oxygen consumption rate, RBC velocity and linear density. For the analytical time scale, the simulated value of $\bar{S}$ at *x* = *L*/2 was employed. Over the investigated range of parameters, the analytical estimates of τ_*CI*_ overestimate the fitted values by at most 12 ms (relative error of ≤ 7.2%). This shows that the variations in τ_*CI*_ that occur in the investigated parameter range can be entirely explained by the dependency of the analytical τ_*CI*_ on μ_*LD*_ and $\bar{S}$. The decay length scale *L*_*CI*_ is equally well predicted by the analytical formulation. Besides, the interaction models have so far employed the Hill equation (Equation 2) to model the equilibrium curve between oxygen and hemoglobin. To examine model robustness, we computed the decay time scale τ_*CI*_ using the Adair equation (Popel, 1989) which is more accurate for *S* ≤ 0.3. The resulting values of τ_*CI*_ are at most 10% smaller at low HS, so the inaccuracy introduced by the Hill equation stays moderate (Figure S6). The structure of the microcirculation in the brain is heterogeneous, and estimates of several relevant parameters such as blood flow rate, capillary spacing and oxygen consumption rate are subject to uncertainties of considerably more than 10%. Therefore, the quantitative errors introduced by the use of the Hill equation are not significant with regard to the analysis of oxygen transport *in vivo*. The main conclusion of this study, namely that diffusive interaction between capillaries can significantly reduce COSH, is not affected by the assumption of the Hill equation.

**Figure 8:**
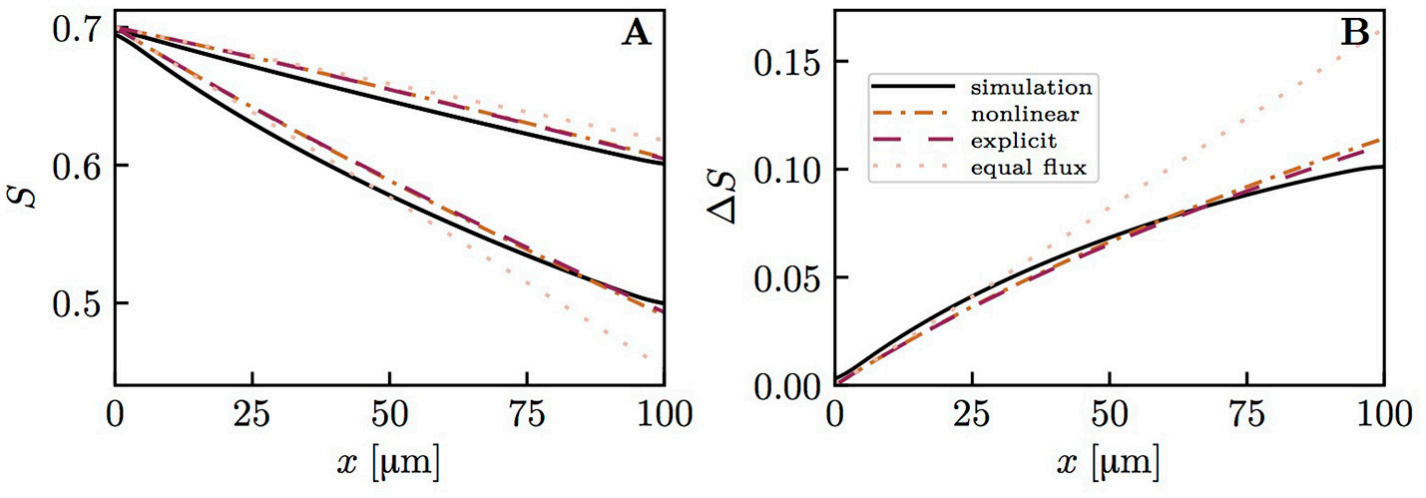
Capillary diffusive interaction with different RBC velocities (*v*_rbc_ = 1.5 and 0.5mm/s). Solid lines: numerical model; dash-dotted lines: nonlinear Krogh-based model; dashed lines: explicit Krogh-based model; dotted lines: equal oxygen flux assumption. **(A)** HS profiles; **(B)** HS difference between both capillary pairs. The simulated final HS difference Δ*S*_*v*_ with *v*_rbc_ = 1.5 mm/s and 0.5 mm/s, respectively, is 41.8% lower than if the oxygen fluxes out of the capillaries are assumed to be equal. The overestimation of Δ*S*_*v*_ by the nonlinear and explicit Krogh-based models is 18.8 and 15.8%, respectively. For smaller differences in *v*_rbc_, the model errors are in the same range.

**Figure 9:**
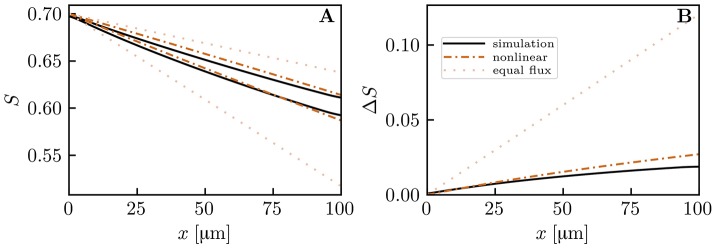
Capillary diffusive interaction with different linear densities (μ_*LD*_ = 0.6 and 0.2). Solid lines: numerical model; dash-dotted lines: nonlinear Krogh-based model; dotted lines: equal oxygen flux assumption. **(A)** HS profiles; **(B)** HS difference between both capillary pairs. The simulated distal HS difference is 84.4% lower than under the assumption of equal oxygen fluxes. The nonlinear Krogh-based model overestimates Δ*S*_*v*_ by 44.4%.

The previous results all assumed the same RBC velocity, flow direction and hematocrit in each capillary. These assumptions are now dropped to further examine model robustness. First, simulations with countercurrent flow instead of concurrent flow were run. Namely, the flows in both pairs of diagonally opposite capillary were set to opposite directions with the same RBC velocity. The HS difference between the venous capillary ends turned out to be practically the same as with concurrent flow (Figure S7). Then, actual CTH was introduced by setting different RBC velocities in the pairs of diagonally opposite capillaries (Figure 8). Similarly, different values of linear density were set in these capillary pairs (Figure 9). The RBC flow was also varied by modifying both RBC velocity and linear density (Figure S8). In all cases, the simulated distal HS difference was considerably lower than under the assumption of equal fluxes. When linear density is heterogeneous, diffusive interaction leads to a stronger reduction of HSH (84.4% in Figure 9) than in the presence of heterogeneous RBC velocities (41.8% in Figure 8). When the RBC flow is varied by modifying both of these parameters, the resulting reduction of HSH (64.5% in Figure S8) falls between the values obtained above. However, the differential equation models were more inaccurate in these cases and significantly overestimated the distal HS difference. Although the interaction models are not as accurate as before with heterogeneous RBC velocities and linear densities, our conclusions about the reduction of HSH still hold in the presence of CTH or hematocrit heterogeneity.

## Discussion

We identified two diffusive interaction mechanisms that cause a large reduction of HSH in capillary networks, developed associated interaction models and validated them using a computational model with individual moving RBCs. The interaction models provide explicit formulas for the reduction of HSH and the associated decay exponents, which gives more insight than a purely computational approach. This work shows that CTH only partially reflects the actual heterogeneity of blood oxygen content and that estimating HSH solely based on CTH may lead to considerable overestimation.

Diffusive interaction between RBCs in a single capillary occurs when two branches with different HS levels converge. This phenomenon is therefore more prevalent in the presence of multiple converging bifurcations along RBC paths. In the mouse cerebral cortex, Sakadžić et al. (2014) estimated the number of capillary branches between arterioles and venules to be 5.9 ± 2.1, with mean segment lengths between 65.6 and 81.4 μm. Since cortical capillary beds have a mesh-like structure (Lorthois and Cassot, 2010; Blinder et al., 2013), each RBC will on average travel through several converging bifurcations. According to the interaction models developed here and the numerical simulations, the standard deviation of HS decays exponentially (Equation 19) with a time scale between 0.15 and 0.21 s (Figure 4). This is slightly below the diffusion time scale given by $r_{t}^{2}/D_{t}=\text{0.22}\;\text{s}$. The time scale τ_*RI*_ was also shown not to depend on the length of the computational domain. Therefore, the RBC interaction time scale is not directly affected by the RBC transit time through the computational domain (although it depends on the RBC velocity). However, τ_*RI*_ can be compared to experimentally measured transit times to examine whether RBC diffusive interaction has enough time to occur while RBCs flow through capillaries. The obtained values of τ_*RI*_ are considerably lower than mean capillary transit times measured using bolus tracking (Gutiérrez-Jiménez et al., 2016) (0.81 ± 0.27 s at baseline, 0.69 ± 0.18 s during activation). These obtained time scales are also smaller than the transit times computed by Schmid et al. (2017) in five analysis layers at different depths in the mouse parietal cortex (0.19 to 0.79 s). Therefore, in the presence of converging bifurcations, this analysis indicates that RBCs spend sufficient time in capillary branches for the HSH to significantly drop.

While the standard deviation of HS in a single capillary generally decreases, our results show that the average value of *S* in a single capillary is not affected by fluctuations of *S*. Since the Po_2_ oscillations caused by the individual erythrocytes do not spread far into the tissue (see values of Δ*r*_osc_ in Figure 5B), tissue oxygenation is likely not adversely affected by the fluctuations in HS observed here. This provides a justification for the oxygen transport models based on a continuum approach for *S* (Goldman and Popel, 1999; Secomb et al., 2000), if the HS downstream of a converging bifurcation is set to the RBC-flow-weighted average of *S* in the upstream branches. Nevertheless, we postulate that the homogenization of *S* in individual vessels is beneficial for oxygen transport, since it reduces the probability of RBCs with very low saturation. Indeed, hypoxia as well as large tissue Po_2_ fluctuations are most likely to occur near vessels with low RBC flow. The homogenization of HS makes it less probable that RBCs with low oxygen content enter such vessels.

Diffusive interaction between capillaries is the second reduction mechanism of HSH that was investigated here. While RBC diffusive interaction primarily occurs downstream of converging bifurcations, capillary diffusive interaction is a more general phenomenon since it does not require the presence of branchings. Our results qualitatively agree with the computations by Popel et al. (1986) in parallel capillary arrays with heterogeneous inlet Po_2_ and erythrocyte velocities. Salathe (2003) performed similar computations in a 5 × 5 capillary array and reported that modeling interaction between functional units smooths out the oxygen concentration differences between capillaries and delays the onset of anoxia. Our results confirm the trends observed in these studies and shed further light on the physiological parameters involved in capillary diffusive interaction.

The range of distances between capillaries (20 to 60 μm) that was examined in our simulations in parallel capillary arrays corresponds to Krogh cylinder radii between 11.3 and 33.8 μm. This includes the mean Krogh radii of the reconstructed MVNs in Fraser et al. (2013) (21.3 ± 2.1 μm to 25.6 ± 3.9 μm) and in Sakadžić et al. (2014) (22.3 ± 1.2 μm to 24.2 ± 2.2 μm) which were obtained by approximating the tissue volume closest to each capillary segment by a cylinder. In our simulations, although the intercapillary distance was tripled, the decay time of the HS difference between parallel capillaries only increased from 0.115 to 0.188 s. This weak dependence on capillary spacing is explained by the formulas for the decay time scale τ_*CI*_ (Equation 34) and the model coefficient *K*_*CI*_ (Equation 29) which only depend on the logarithm of the ratio between the mean Krogh radius and the capillary endothelium radius. These time scale values are lower than the decay time scale τ_*RI*_ for RBC diffusive interaction and also significantly smaller than the capillary transit times reported above. Additionally, our theoretical analysis showed that the HS difference between parallel capillaries decays exponentially (Equation 32). This provides compelling evidence that diffusive interaction between capillaries is a strong mechanism for the reduction of HSH at the scale of neighboring capillaries. Its occurrence regardless of the presence of converging bifurcations suggests that this is a more general phenomenon than RBC diffusive interaction. Finally, unlike the latter mechanism, capillary diffusive interaction strongly influences the mean HS drop along microvessels and thus affects more significantly tissue oxygenation.

Having shown the importance of diffusive interaction mechanisms, it is natural to ask up to which length scale they can act. While RBC interaction is confined to single capillaries, hence very local, it is not evident how far reaching capillary interaction can be. The weak dependence of the decay time scale τ_*CI*_ on capillary spacing (Equation (34) and Figure 7C) suggests that this oxygen transfer mechanism can be relevant for capillary distances above 50 μm, which is higher than typical inter-capillary spacings in the cerebral cortex (Tsai et al., 2009) or muscles (Ellsworth et al., 1988). To determine the maximal length scale of capillary diffusive interaction, it will be necessary to understand how capillaries with irregular spacings (see Hoofd and Turek, 1996) influence each other's supplied tissue regions. We propose the concept of diffusive interaction length scale as a tool to compare CTH and COSH. Our results provide strong evidence that HSH on the scale of the inter-capillary distance is efficiently damped by diffusive interaction. Whether this still holds for medium or large-scale HSH is an open question. Its answer is essential to assess the consequences of disturbed capillary flow patterns on oxygen transport based on their spatial scale.

The models for RBC and capillary diffusive interactions enable the computation of mean HS and its heterogeneity in single and parallel vessels, respectively. Previously, the relation between CTH and oxygen extraction fraction was studied by Jespersen and Østergaard (2012) using the Bohr-Kety-Crone-Renkin equation and extended by Angleys et al. (2015) Their approach does not account for the heterogeneity of hematocrit, vessel size and spacing which occurs in capillary networks. Our approach takes into account each of these parameters and shows that distal HS is not only a function of the transit time (Equation 11). In particular, it includes the influence of hematocrit which was shown to have a paramount influence on tissue Po_2_ (Lücker et al., 2017). Another major advance in this work is the modeling of interaction between capillaries through the presence of converging bifurcations and diffusive oxygen transfer. Instead of dealing with idealized distributions of capillaries with independent supplied tissue regions, the models developed here lay the ground for a refined analysis of HSH in realistic MVNs. This is an essential step to assess the actual consequences of CTH on oxygen transport and availability in the microcirculation.

The diffusive interaction models consist in ordinary differential equations that can be easily integrated. The simplifications done in their derivation give rise to slight inaccuracies with respect to the computational model. The errors of the RBC and capillary diffusive interaction models are ≤ 4% (Figure S3) and ≤ 8% (Figure S4), respectively. The main sources of inaccuracy are the linearization of the terms d*P*_eq_/d*S* (Equation 15) and *P*_eq_ (Equation 18) as well as the neglect of axial diffusion. For capillary diffusive interaction, the approximation of the supplied tissue region by a cylinder (Figure 2B) and Equation (27) are additional sources of inaccuracy. Since these errors originate in the reduction of nonlinearly coupled partial differential equations to ordinary differential equations, these inaccuracies are a very moderate price to pay.

The limitations to our diffusive interaction models include the oxygen-independent metabolic consumption term *M*_0_, which provides an analytical solution to the radial oxygen transport equation in the tissue. At low tissue Po_2_, metabolic oxygen consumption modeled with Michaelis-Menten kinetics may produce higher tissue Po_2_ and thus influence the supplied tissue cylinder radii in capillary diffusive interaction. However, the choice of this consumption model over constant oxygen consumption only has a limited influence on the oxygen profiles in a cylindrical geometry (Grimes et al., 2014). Similarly, metabolic oxygen consumption is expected to vary spatially, as suggested by the depth-dependent neuron density in the mouse cerebral cortex (Tsai et al., 2009). Heterogeneous oxygen demand may result in an increase or a decrease in COSH, depending on its covariation with blood flow and vessel spacing. As a further limitation, the Bohr effect was not modeled. Since the derived equations for the evolution of HSH (Equations (19) and (32)) and the characteristic scales *L*_*CI*_ and τ_*CI*_ (Equations (33) and (34)) explicitly depend on the derivative of the equilibrium curve *P*_eq_(*S*), its shift may influence the reduction mechanisms of HSH. Likewise, the inaccuracy of the Hill equation at low HS also has an influence, albeit in a limited way (Figure S6). Finally, since we focused on physiological parameters typical for the cerebral cortex, myoglobin-facilitated diffusion of oxygen in tissue was not considered. Its inclusion into our models would decrease the time scales for capillary diffusive interaction (Equations (29) and Equation (34)).

This study extensively investigates HSH in a Krogh cylinder geometry and parallel capillary arrays. Thus, our conclusions are currently limited to tissues with approximately parallel and straight capillaries such as striated muscles. The next step is to verify whether our theoretical predictions hold when blood vessels are interconnected, tortuous and have variable spacings. In a follow-up article, we are going to present simulations of oxygen transport in microvascular networks from the mouse somatosensory cortex. The distribution of capillary transit times will be compared to that of outflow HS. Then, the interaction models will be used to quantify how much diffusive interaction reduces COSH. The spatial scale up to which diffusive interaction acts also requires further investigation. This could be performed using parallel capillary arrays with more vessels or large realistic microvascular networks. In addition to numerical simulations, experimental data are needed to confirm our theoretical predictions. To achieve this, measurements of CTH based on bolus tracking (Gutiérrez-Jiménez et al., 2016) could be combined with intravascular measurements of capillary Po_2_ using two-photon phosphorescence laser microscopy (Finikova et al., 2008).

In conclusion, this study lays the theoretical basis for the analysis of HSH in MVNs. It is a substantial improvement over previous approaches in the brain that were limited to independent, identical capillaries without branchings. Models for RBC and capillary diffusive interactions were developed and successfully validated using a detailed computational model in simplified geometries. The following conclusions can be drawn: (1) diffusive interaction leads to a strong reduction of small-scale HSH caused by CTH or other factors; (2) HSH can arise in the absence of CTH, for instance due to differences in hematocrit or supplied tissue volume; (3) CTH influences COSH, but does not determine it. Thus, this modeling work is a major step to better understand the actual effects of CTH on blood oxygen content. This has potential implications in the study of all conditions where capillary dysfunction and CTH are thought to be involved, such as Alzheimer's disease (Østergaard et al., 2013a), stroke (Østergaard et al., 2015), traumatic brain injury (Østergaard et al., 2014a) and ischemic heart disease (Ostergaard et al., 2014b).

## Author contributions

AL conceived of the study, developed the theoretical models, implemented the algorithms, ran the simulations, interpreted the data and drafted the manuscript. TS contributed the initial idea for the study. BW and PJ conceived of the study and participated in its design.

### Conflict of interest statement

The authors declare that the research was conducted in the absence of any commercial or financial relationships that could be construed as a potential conflict of interest.

## Abbreviations

COSH: capillary outflow saturation heterogeneity
CTH: capillary transit time heterogeneity
HS: hemoglobin saturation
HSH: hemoglobin saturation heterogeneity
RBC: red blood cell.
