# Supplementary material for: The Relation Between Capillary Transit Times and Hemoglobin Saturation Heterogeneity. Part 1: Theoretical Models
Source: Front Physiol. 2018 Apr 26;9:420. doi: 10.3389/fphys.2018.00420 (PMC5932636; doi:10.3389/fphys.2018.00420)
Supplement: Supplementary file 1 [file Data_Sheet_1.PDF]

Supplementary Material:
Article Title

Adrien Lücker [1,*], Timothy W. Secomb [2], Bruno Weber [3] and Patrick Jenny [1]

*Correspondence:
Adrien Lücker
luecker@ifd.mavt.ethz.ch

1 SUPPLEMENTARY FIGURES

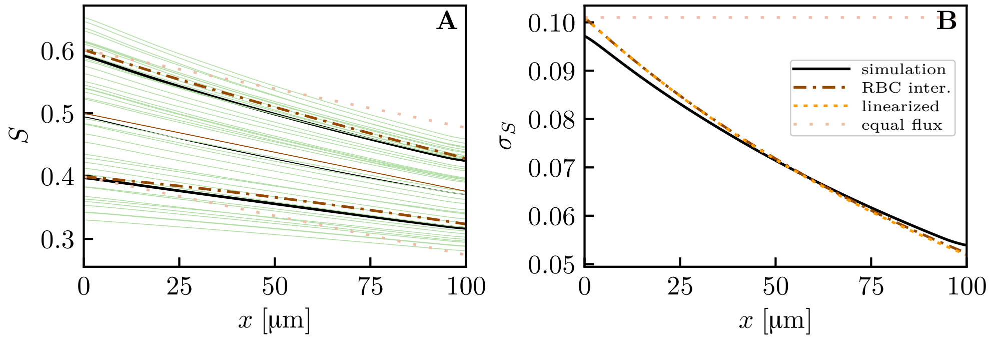

Figure S1. Hemoglobin saturation profiles with a uniform distribution of S_a. The simulation was run in the geometry with four parallel capillaries (Figure 2A) with a uniform inflow distribution of hemoglobin with mean $S = 0.5$ and standard deviation $\sigma_S = 0.1$ in each capillary. $\mu_{LD} = 0.3$; $v_{\mathrm{rbc}} = 1.0\,\mathrm{mm/s}$. **(A)** Thin green lines: individual RBCs from the numerical simulation; thick solid lines: mean $S \pm \sigma_S$; dash-dotted lines: RBC interaction model; dotted lines: assumption of equal oxygen flux out of the RBCs. **(B)** See legend of Figure 3. The fitted value of K_{RI} was $9.28\,\mathrm{mmHg\,\mu m\,s/(\mu m^3\,O_2)}$, whereas it was $9.37\,\mathrm{mmHg\,\mu m\,s/(\mu m^3\,O_2)}$ for a two-dimensional simulation with the same input parameters and alternating inlet values $S = 0.6$ and 0.4. Based on this close agreement, it is justified to investigate our models for RBC diffusive interaction in a two-dimensional cylindrical domain and with alternating inlet values for HS.

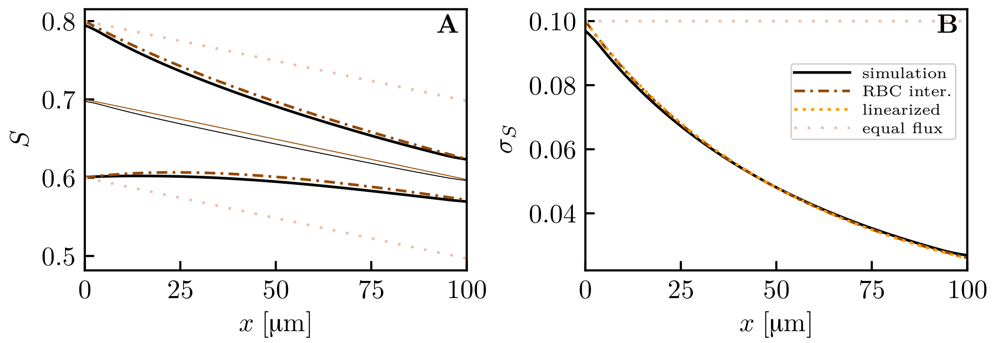

Figure S2. Hemoglobin saturation profiles with alternating inlet values in the cylindrical geometry with phyiosological parameters for a working muscle. The simulation was run with parameters for hamster retractor muscle as in Eggleton et al. (2000) (albeit without myoglobin-facilitated diffusion in the tissue). The RBC velocity was set to $v_{\mathrm{rbc}} = 0.465\,\mathrm{mm/s}$ which is equal to five times the mean velocity in resting muscle. The working muscle consumption $M_0 = 1.62 \times 10^{-3}\,\mathrm{\mu m^3\,O_2\,\mu m^{-3}\,s^{-1}}$ was used. $V_{\mathrm{rbc}} = 69.3\,\mathrm{\mu m^3}$, $L_{\mathrm{rbc}} = 8.16\,\mathrm{\mu m}$, $\mu_{LD} = 0.516$, $r_p = 1.8\,\mathrm{\mu m}$, $r_w = 2.1\,\mathrm{\mu m}$, $r_t = 14.9\,\mathrm{\mu m}$, $P_{50} = 29.3$, $n = 2.2$. Solid lines: numerical simulation; dash-dotted lines: RBC interaction model; dotted line: linearized RBC interaction model; pale dotted lines: assumption of equal oxygen flux out of the RBCs. **(A)** HS profile of heterogeneously saturated RBCs (thin line: average). **(B)** Standard deviation profile of S.

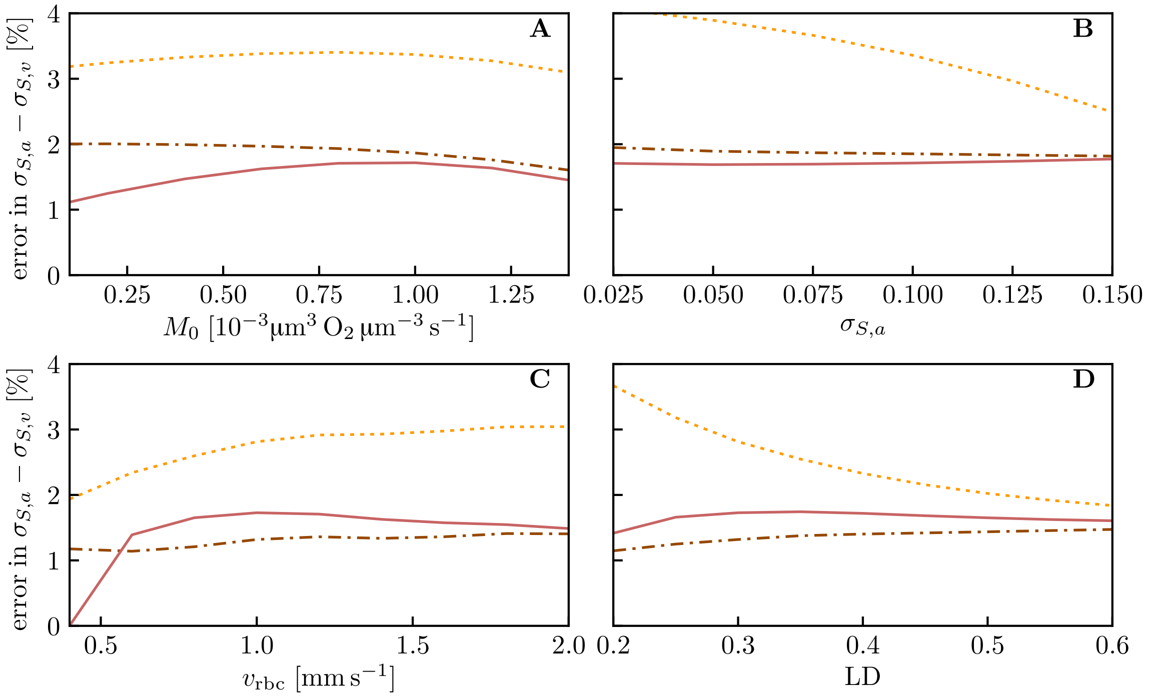

Figure S3. Relative errors from the RBC diffusive interaction models compared to the numerical simulations. The relative error in the drop in σ_S across the capillary is defined as $(\sigma_{S,v,\mathrm{simul}} - \sigma_{S,v,\mathrm{model}})/(\sigma_{S,a,\mathrm{simul}} - \sigma_{S,v,\mathrm{simul}})$. Dash-dotted lines: RBC interaction model; dotted lines: linearized model; solid lines: exponential fit to the numerical results. **(A)** oxygen consumption rate; **(B)** standard deviation of HS at the inlet; **(C)** RBC velocity; **(D)** linear density.

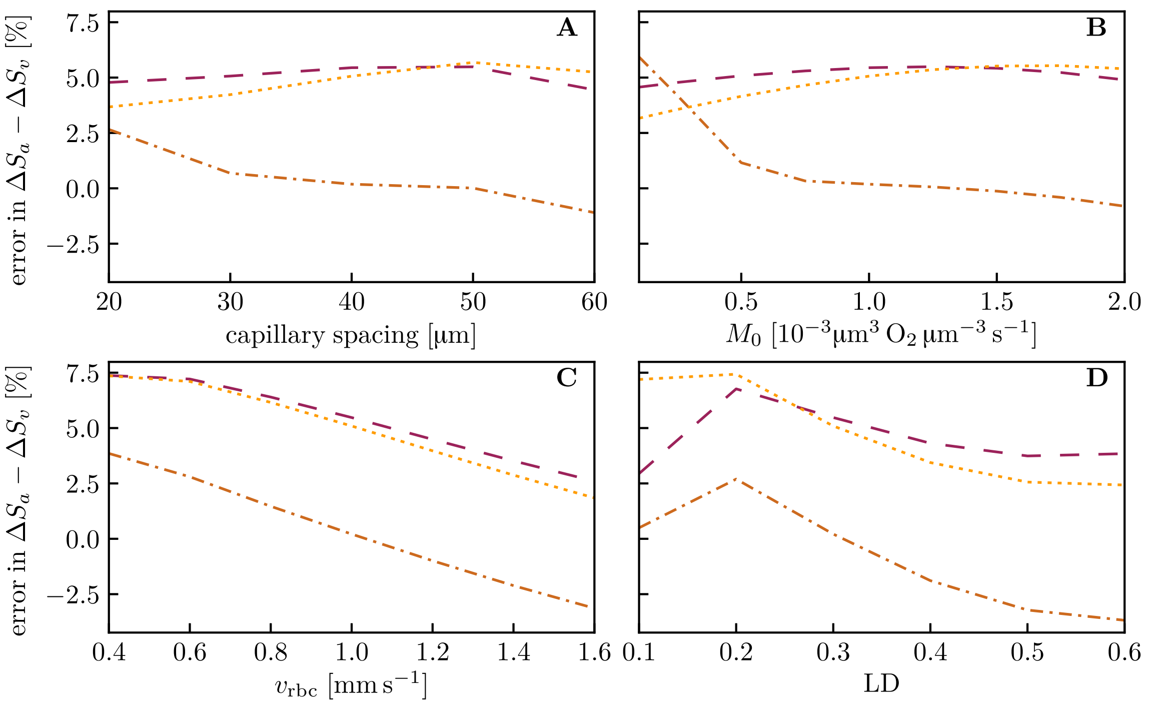

Figure S4. Relative errors from the capillary diffusive interaction models compared to the numerical simulations. The relative error of the drop in HS difference across the capillary is defined as $(\Delta S_{v,\mathrm{simul}} - \Delta S_{v,\mathrm{model}})/(\Delta S_{a,\mathrm{simul}} - \Delta S_{v,\mathrm{simul}})$. Dash-dotted lines: nonlinear Krogh-based model; dashed lines: explicit Krogh-based model; dotted lines: linearized model for ΔS. **(A)** distance between capillaries; **(B)** oxygen consumption rate; **(C)** RBC velocity; **(D)** linear density.

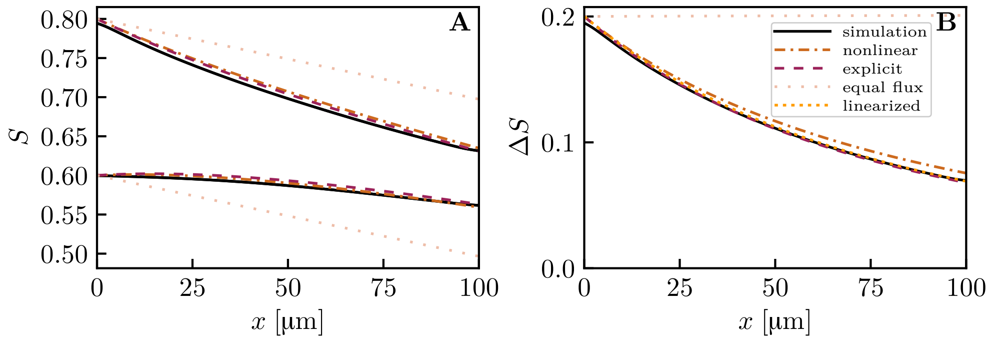

Figure S5. Hemoglobin saturation profiles in parallel capillaries with physiological parameters for a working muscle. The parameters are the same as in Figure S2. The distance between the capillaries was set to 26.4 µm, which is equivalent to a tissue radius of 14.9 µm. Solid lines: numerical simulation; dash-dotted lines: nonlinear Krogh-based model; dashed lines: explicit Krogh-based model; dotted line: linearized model for ΔS; pale dotted lines: equal outflux assumption. **(A)** HS profile in both model capillaries. **(B)** HS difference between both capillaries. The slight overestimation of ΔS (8.5%) at the distal end of capillary by the nonlinear Krogh-based model is due to the intravascular resistance coefficient. The employed value of K_{IV} was obtained from previous simulations (Lücker et al., 2017) with the same parameters as in Table 1. Since the capillary radius is lower with the parameters from (Eggleton et al., 2000), this leads to a slight overestimation of K_{IV} which causes ΔS to be too high (see Eq. (23)).

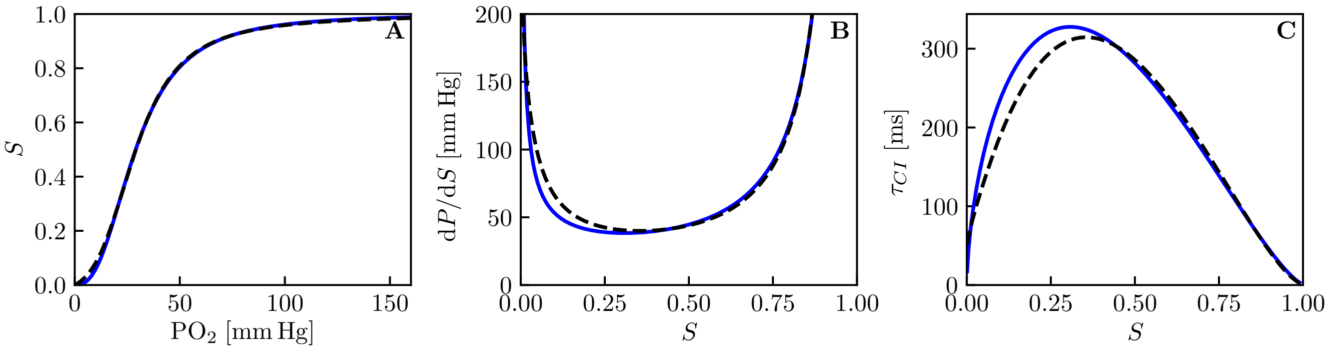

Figure S6. Influence of the oxygen-hemoglobin dissociation curve on the decay time scale τ_{CI} for capillary diffusive interaction. The Hill equation and the Adair equation (Popel, 1989) were employed to parameterize the oxygen-hemoglobin dissociation curve for human blood. Coefficient values were taken from Winslow et al. (1977). $\mu_{LD} = 0.3$. **(A)** Oxygen-hemoglobin dissociation curve. **(B)** Derivative $\frac{\mathrm{d}P}{\mathrm{d}S}$ of the dissociation curve. **(C)** Decay time scale τ_{CI}. Solid line: Hill equation with $P_{50} = 29\,\mathrm{mmHg}$ and $n = 2.6$; dashed line: Adair equation.

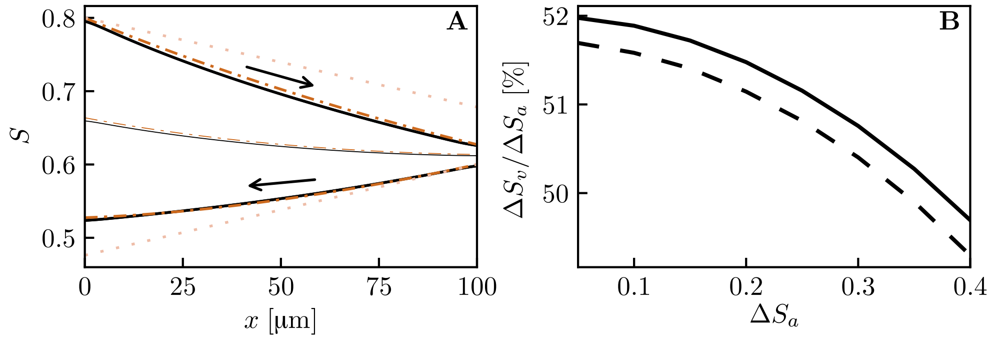

Figure S7. Capillary diffusive interaction with concurrent and countercurrent flow. $\mu_{LD} = 0.3$; $v_{\mathrm{rbc}} = 1.0\,\mathrm{mm/s}$. **(A)** HS profiles with countercurrent flow and inlet values $S = 0.8$ and 0.6, respectively. Solid lines: numerical model; dashed lines: Krogh-based model; dotted lines: equal oxygen flux assumption. Thick lines: values in individual capillaries; thin lines: averaged value over all capillaries. The arrows indicate the flow direction. **(B)** percentual drop in ΔS from the arterial to the venous side of the capillaries. Solid line: concurrent flow; dashed line: countercurrent flow.

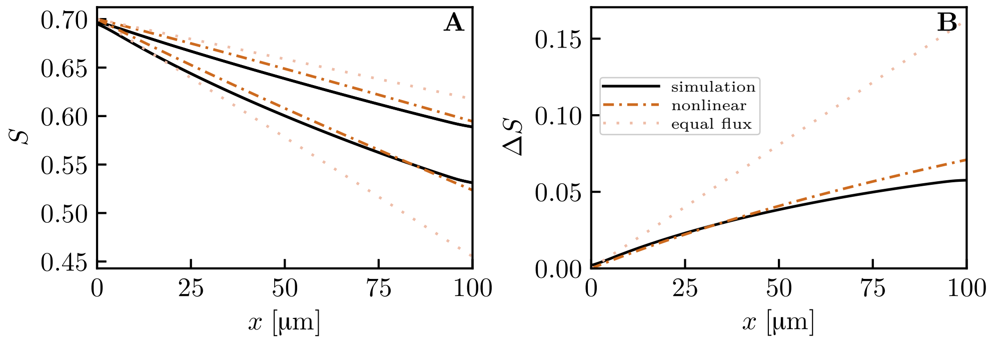

Figure S8. Capillary diffusive interaction with different RBC flows ($v_{\mathrm{rbc}} = 1.22\,\mathrm{mm/s}$, $\mu_{LD} = 0.367$ and $v_{\mathrm{rbc}} = 0.707\,\mathrm{mm/s}$, $\mu_{LD} = 0.212$, respectively). The values of RBC velocity and linear density were set to obtain the same RBC flow values as in Figures 8 and 9. Solid lines: numerical model; dash-dotted lines: nonlinear Krogh-based model; dotted lines: equal oxygen flux assumption. **(A)** HS profiles; **(B)** HS difference between both capillary pairs. The simulated final HS difference ΔS_v is 64.5% lower than if the oxygen fluxes out of the capillaries are assumed to be equal. The nonlinear Krogh-based model overestimates ΔS_v by 18.8%.

REFERENCES

Eggleton, C., Vadapalli, A., Roy, T., and Popel, A. (2000). Calculations of intracapillary oxygen tension distributions in muscle. *Math Biosci* 167, 123–143. doi:10.1016/s0025-5564(00)00038-9

Lücker, A., Secomb, T. W., Weber, B., and Jenny, P. (2017). The relative influence of hematocrit and red blood cell velocity on oxygen transport from capillaries to tissue. *Microcirculation* 24, e12337. doi:10.1111/micc.12337

Popel, A. (1989). Theory of oxygen transport to tissue. *Crit Rev Biomed Eng* 17, 257–321

Winslow, R. M., Swenberg, M., Berger, R. L., Shrager, R. I., Luzzana, M., Samaja, M., et al. (1977). Oxygen equilibrium curve of normal human blood and its evaluation by adair's equation. *Journal of Biological Chemistry* 252, 2331–2337
